# Artificial Intelligence Vision Methods for Robotic Harvesting of Edible Flowers

**DOI:** 10.3390/plants13223197

**Published:** 2024-11-14

**Authors:** Fabio Taddei Dalla Torre, Farid Melgani, Ilaria Pertot, Cesare Furlanello

**Affiliations:** 1 Department of Information, Engineering and Computer Science, University of Trento, 38122 Trento, Italy; 2 Antares Vision S.p.A, 25039 Travagliato, Italy; 3 LIGHT Center, 25123 Brescia, Italy

**Keywords:** deep neural networks, computer vision, precision farming, edible flowers

## Abstract

Edible flowers, with their increasing demand in the market, face a challenge in labor-intensive hand-picking practices, hindering their attractiveness for growers. This study explores the application of artificial intelligence vision for robotic harvesting, focusing on the fundamental elements: detection, pose estimation, and plucking point estimation. The objective was to assess the adaptability of this technology across various species and varieties of edible flowers. The developed computer vision framework utilizes YOLOv5 for 2D flower detection and leverages the zero-shot capabilities of the Segmentation Anything Model for extracting points of interest from a 3D point cloud, facilitating 3D space flower localization. Additionally, we provide a pose estimation method, a key factor in plucking point identification. The plucking point is determined through a linear regression correlating flower diameter with the height of the plucking point. The results showed effective 2D detection. Further, the zero-shot and standard machine learning techniques employed achieved promising 3D localization, pose estimation, and plucking point estimation.

## 1. Introduction

High-value crops are non-traditional food crops with a higher market value than staple crops. Examples include vegetables, foliage, condiments, spices, and edible flowers. Among them, edible flowers have gained growing consumer interest, leading to an increasing trend in production [1]. Indeed, consumers’ interest in edible flowers is moving from a simple plate ornament to real food recognized as a source of micro-nutrients, including antioxidants and vitamins. Therefore, edible flowers are a noteworthy opportunity for farmers to generate income, especially in the floriculture sector [2,3,4]. Unfortunately, the high labor demand, particularly during harvesting, which is often carried out throughout the year, is a critical issue [5]. Further, edible flowers require high aesthetical standards and a gentle cut. This is a labor-intensive practice that poses economic challenges for farmers and increases the difficulty of finding qualified employers [6]. Therefore, the robotic harvesting of edible flowers could represent a socio-economically sustainable alternative to hand picking. Although edible flowers’ harvesting is well suitable for robotization because they are commonly cultivated in highly technological greenhouses, few solutions have been studied [5,7].

Few robotic harvesting attempts of high-value crops have been made. A robotic harvester for ridge-planted strawberries utilized a customized version of YOLOv3 for real-time fruit localization and picking point detection [8]. This method aims to minimize end-effector movements, thereby preventing damage to neighboring fruits. Another solution focused on an autonomous harvester for high-quality tea, addressing challenges analogous to edible flower picking. The key components of this approach are a detection module, plucking point localization, and motion planning. In particular, the tea harvester prototype leveraged the merging of depth images by RGB-D cameras, which provide both depth and color data: RGB-D data were used to extract the tea shoot growth direction, determining the plucking point as the intersection between the main axis and the plucking plane [9]. Subramanian and colleagues [10] developed a robotic saffron flower harvesting system, also including methods for detection, localization, pose, and plucking point estimation. Interestingly, the developed solution can collect one flower every 6 s, where one second is allocated for image processing purposes, and the remaining 5 s for the harvesting operation.

A limitation of studies on robotic harvesting is their focus on a single species or, in many instances, a specific variety of the crop of interest. The objective of our work was to extend beyond this narrow focus by developing an artificial intelligence (AI)-based vision framework supporting robotic harvesting that is adaptable across different species and varieties, minimizing the need for tailoring specific solutions to each of them. Our specific objectives were as follows:

- Develop a novel combination of AI modules for detection, pose estimation, and plucking point estimation modules for multi species picking;
- Focus on machine learning methods that require very limited or no novel training data (also called few-shot and zero-shot approaches) to ensure high-quality results without requiring extensive data collection and annotation;
- Novel plucking point estimation method through indirect inference, leveraging a dataset of collected flower measurements.

The overall approach also aimed to mitigate the risk of overfitting to specific variety or dataset characteristics. Edible flowers were selected as a case study due to the lack of developed solutions and their significant variations in shape and color.

## 2. Materials and Methods

The case study was implemented in a commercial greenhouse located in Mira (VE), Italy, owned by L’Insalata dell’Orto s.r.l. [11], a leading company in the edible flower market specialized in the cultivation, packaging, and distribution of fresh vegetables, salads, and 17 species/varieties of edible flowers. Plants were grown on benches (70 cm high, 110 cm wide, and about 50 m long), which is the most commonly used in greenhouse floriculture. This layout offers several advantages for robotic harvesting, including a consistent height from the ground and a straight-line configuration, which enhances the uniformity of the collected data. Additionally, edible flowers are harvested daily and, during peak periods, multiple times a day. This characteristic is particularly beneficial for robotic harvesting, as it minimizes the issues of overlap and occlusion; overlapping flowers are picked first, revealing those hidden beneath. Furthermore, the repeated and random scanning of the crop reduces the likelihood of encountering the same occlusions in different harvests.

**Figure 1:**
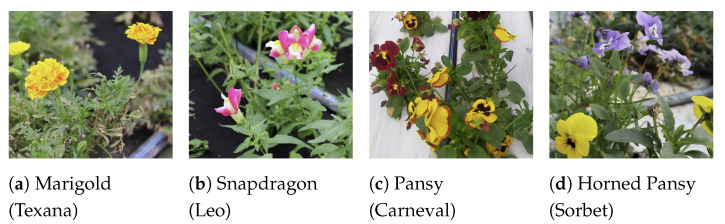
Sample images of the four edible flowers considered in the experiments.

The experiments focused on four species (Figure 1) that represent a substantial portion of the company’s production and exhibited distinct characteristics on both flower and plant morphology, providing a diverse sample for testing the implemented techniques: marigolds (*Tagetes patula* cv Texana), snapdragon (*Antirrhinum majus*, cv Leo), pansy and horned pansy (*Viola* x *wittrockiana* cv Carneval and *Viola cornuta* cv. Sorbet, respectively). By considering different types of flowers, this work aimed to test the generalization capability of the framework on flowers presenting various poses. For instance, snapdragon flowers tend to lean to one side, while marigolds and pansies generally exhibit an upright posture. The study also examined flowers of different sizes, with snapdragons having an average diameter of 28 mm, marigolds 36 mm, and pansies reaching up to 60 mm. Since this study focused on overall trends and conclusions derived from the results, pose estimation and plucking point estimation figures are provided for two flower types, marigold and snapdragon, which present contrasting characteristics. The two varieties of pansies are treated as a single flower type, collectively referred to as “pansy”.

### 2.1. Data Acquisition and Annotations

**Figure 2:**
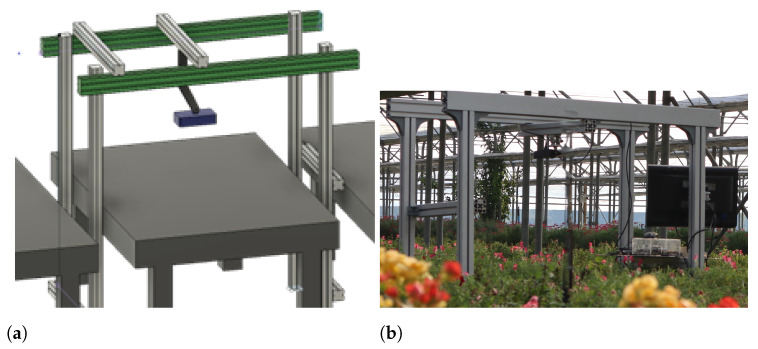
Acquisition cart: (**a**) 3D blueprint of the cart; (**b**) example of acquisition in the greenhouse.

Given the cultivation layout, a straddle cart was assembled using modular aluminum profiles, presenting a width of 120 cm, an overall height of 175 cm, and a pitch between the front and rear wheels of 80 cm (Figure 2a,b). This design facilitates the smooth movement of the conveyance across the cultivation benches, enabling the acquisition of data from a vertical position relative to the crops.

**Figure 3:**
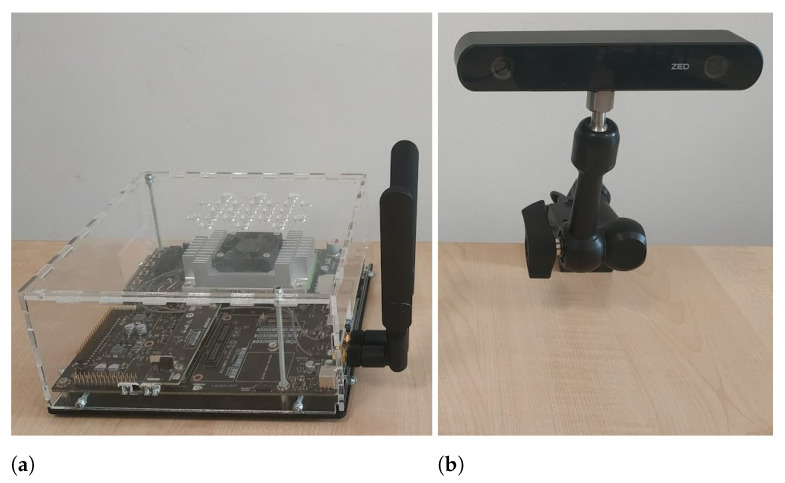
Jetson TX2 module (**a**) and Zed2i (**b**) integrated in the data acquisition setup.

The conveyance was equipped with a stereo camera to capture 2D and 3D images of the crop. Two versions of Stereolabs (San Francisco, CA, USA) RGB-D cameras were tested for assessing the generalization capabilities and robustness of the implemented models: Zed 2 and Zed 2i (Figure 3b). The Zed 2 stereo camera provided a field of view of up to 120° with two 4M-pixel 2.1 mm sensors in a native 16:9 format, while Zed 2i offered an 81° field of view with two 4 mm sensors and additional polarized filters.

Zed Cameras [12], being RGB-D stereo cameras, generate a depth point cloud that associates a depth measure with each captured pixel. This point cloud is derived through Neural Stereo Depth Sensing technology. To support this functionality, the cart is also equipped with a Jetson TX2 [13] (Figure 3a), a comprehensive system-on-module developed by Nvidia (Santa Clara, CA, USA) to execute AI models at the “edge”, which is an operation set-up in real time. The Jetson TX2 module is utilized for computing images and point clouds of the crop through a Python GUI combined with Stereolab’s Python programming interface.

**Figure 4:**
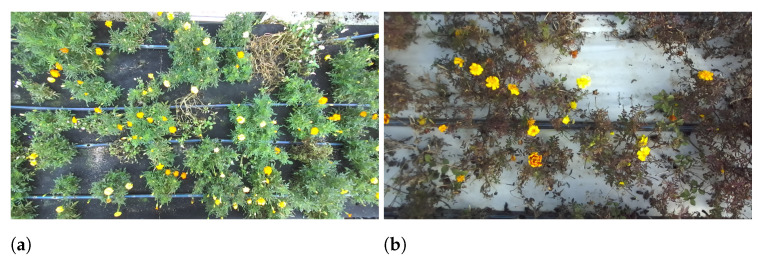
Comparison of marigold flower conditions: (**a**) July vs. (**b**) November.

In order to introduce environmental variability into the dataset, particularly concerning the impact of cultivation conditions (Figure 4), two data collection campaigns were carried out on 22 July and 17 November 2023. The flowers were cultivated in a commercial greenhouse where environmental conditions were partially controlled (i.e., temperature within the range of optimal/suboptimal conditions for the plant, sufficient irrigation), and the flowering induction of various species experienced seasonal changes mainly based on the photoperiod and intervals of suboptimal temperature. For example, the density of flowers for a square meter of the reference varieties of marigold and sSnapdragon can range from 3 to 50 flowers. In both campaigns, images of marigold and snapdragon were collected, while pansy was exclusively gathered during the second campaign.

A total of 134 images containing 2443 flowers overall were collected in the FloraDet dataset for model development and validation. Manual annotation based on the online annotation tool Roboflow (Des Moines, IA, USA) [14] was employed (one unique trained operator). The *FloraDet* dataset is described in Table 1. According to the commercial standard quality, an edible flower is deemed ripe and ready for picking when it has achieved 70–80% of overall opening. Accordingly, only flowers meeting this criterion were annotated to ensure that the system detected only ripe flowers.

**Figure 5:**
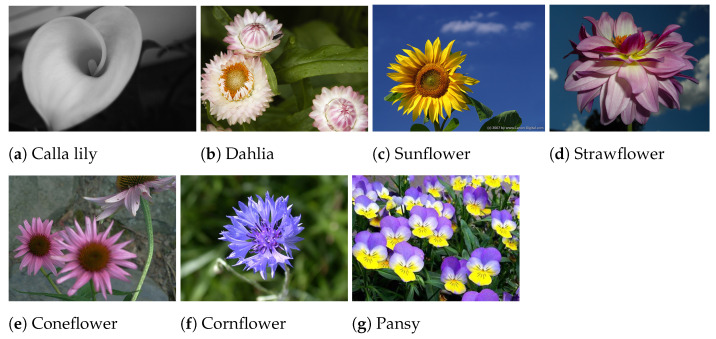
Examples of the different flowers in the D0 dataset from ImageNet and Kaggle.

For preliminary testing and fine-tuning, we established an additional *D0* flower dataset by merging images from two distinct sources: ImageNet [15] and Kaggle [16]. Notably, the D0 image dataset comprised flower varieties different from those in FloraDet (Figure 5). Given the well-established transfer learning capabilities of neural networks, the D0 dataset was utilized for an initial fine-tuning process of the AI model. Specifically, we sourced a set of 1033 images, including flowers from ImageNet, along with their corresponding annotated bounding boxes. This involved retrieving images and annotations from ImageNet Large Scale Visual Recognition Challenge 2010 (ILSVRC2010) [17] of six different synsets (Table 2).

The D0 dataset was further enriched with images pertaining to pansy flowers after an empirical observation during training: challenges in classifying pansy flowers, likely due to their significant variability in color and shape. In addition, pansies were underrepresented in the FloraDet dataset, contributing to the challenges faced by the model. A total of 232 images of pansy flowers were obtained from two online datasets: Flower-299 [18] and Flower Color Images [19], both from Kaggle. Since these datasets were originally designed for image classification and lacked bounding boxes, two distinct annotation processes were implemented. The first involved autonomous annotation via zero-shot detection using the open-vocabulary object detection network OWL-ViT [20]. A portion of about 43% (*n* = 101) images were first annotated with OWL-ViT; the remaining *n* = 131 images were manually annotated using the Roboflow annotation tool. This manual annotation process was also beneficial in identifying and removing duplicate images.

### 2.2. Vision Module Pipeline

**Figure 6:**
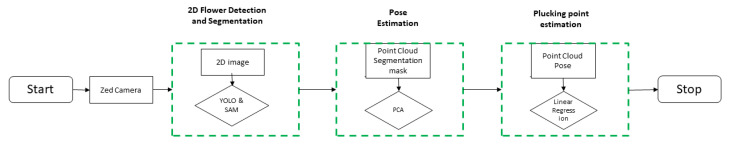
Main workflow for the AI-based vision pipeline. The dashed-green bounding boxes represent the three main modules, indicating inputs (black rectangles) and main implemented methods (black rhomboids).

The operational workflow of the pipeline is composed of three individual modules: *Flower Detection*, *Pose Estimation*, and *Plucking Point Estimation* (Figure 6).

Each module operates in cascade, receiving the output of the preceding one as input. Each module is explained and described in the following sections. Specifically, in the preliminary phase described in this work, after the camera slides over the crop, an image is captured and then undergoes processing by all the building blocks within the pipeline.

#### 2.2.1. 2D Detection and Segmentation

To achieve rapid, reliable, and high-quality results, we explored cutting-edge AI architectures to detect ripe flowers and then utilized segmentation to precisely isolate them within the environment. A 2D flower detection was obtained by Ultralitics’s “You Only Look Once” YOLOv5 [21]. Specifically, YOLO is an architecture proposed in 2016 by Redmon et al. [22], which approaches the detection problem as a regression problem. This approach allows the architecture to process the whole image at once (hence its name), making the network fast and capturing global and contextual information that can aid in the detection process. Briefly, the entire image is fed to the network, which divides the image into a grid of cells. For each of these cells, a set of bounding boxes, along with confidence scores and class probabilities, are predicted. The final detection is obtained through Non-Maximum Suppression, reducing the number of overlapping boxes.

Despite the fundamental idea remaining the same, many versions of YOLO have been developed [23]. There exist YOLOv2, YOLOv3, YOLOv4, the one exploited in this work, which is YOLOv5, and subsequent versions. Roughly, the architecture of this network can be schematized as follows in terms of backbone (from input to feature extraction), neck (feature preprocessing), and head (prediction) components: firstly, CSP-Darknet acts as the backbone, responsible for the feature extraction process. The results of this layer are then passed to the neck, which is based on PANnet and combines image features, passing them to the head. The head basically constitutes convolution layers responsible for generating predictions and bounding boxes [24]. Although more recent and sophisticated versions are available such as YOLOv8, this architecture is quite similar to YOLOv5, which, after training on a custom dataset, proved to be sufficient as a component of our pipeline. YOLOv8 [25] also offers semantic segmentation, but this feature was not utilized to avoid additional data annotation and to prioritize zero-shot possibilities. Therefore, the latest release, YOLOv8, was not used in this work.

Different variants of the same version of YOLOv5 are available; the difference lies in the depth and layer multiple of the head. In the model YoloV5l, the one employed in this work, these two parameters are set to 1, which means the standard depth and layer channels are used as a trade off for lightweight versions with respect to heavyweight versions. When the two parameters are small, the network is lighter and faster in inference, but at the same time, it decreases its accuracy, which is vice versa for heavier versions.

Specifically, in this work, YoloV5l pre-trained on COCO underwent fine-tuning initially on the D0 dataset and subsequently on the collected images, with a specific focus on the left image. The outcome of the fine-tuned YOLO version, referred to as FLOLO, was then used as input for SAM, generating a binary mask for the precise segmentation of flowers in the image.

For segmentation, we leveraged the zero-shot capabilities of the Segmentation Anything Model (SAM) [26]. Additionally, SAM was selected for its seamless integration with YOLO, as its bounding boxes can serve as input prompts for SAM.

SAM is a new model for image segmentation proposed by Kiriloc et al. [26], which has already become a foundational model for image segmentation. Like other foundational models such as DALL-E or GPT-3, SAM’s capabilities can be easily exploited to perform new tasks just by providing a simple prompt [27]. SAM’s architecture is as straightforward as efficient: the input image is processed by a pre-trained Vision Transformer (ViT), and the resulting image embedding is then passed to the mask decoder, which is a customized Transformer decoder block, together with the input prompt processed by the prompt decoder. The peculiarity of this architecture is that it is capable of managing prompts in different forms: text, points, boxes, but also other masks.

As for YOLO, different pre-trained configurations depending on the version of ViT are available. In this work, the model based on ViT huge [28] was applied.

Specifically, an image, along with the corresponding YOLO predicted bounding boxes, is inputted into SAM, generating a binary mask as output that effectively separates pixels related to flowers from other objects.

#### 2.2.2. Pose Estimation

Pose estimation is one of the most pivotal and delicate components of robotic harvesting since precise and secure picking is ensured only by precise and secure pose estimation [29]. For approximately spherical fruits, like tomatoes, this problem is addressed by matching a predefined 3D model with the point cloud of the target fruit. This approach seamlessly identifies the perfect picking point for the fruit. Unfortunately, this approach cannot be applied to more complex-shaped fruits, as in the case of flowers [30].

**Figure 7:**
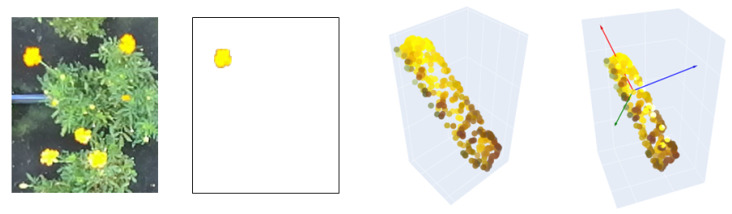
Flower pose estimation for steps. From left to right: input image, single flower cutout trough SAM, derived isolated point cloud in the 3D space, and PCA-based point cloud analysis.

Similar to previous steps, our approach focuses on minimizing the need for extensive training, starting with binary segmentation. In this process, the entire point cloud, comprising 921,600 points, provided by the ZED SDK is used. Since the ZED SDK output is already preprocessed and denoised, the point cloud can be used directly. Notably, segmentation is applied to the left image. As the 3D point cloud from the ZED camera aligns with the left image, we can selectively extract flower-related points by masking the 3D point cloud with the 2D segmentation. For pose estimation, a simple Principal Component Analysis (PCA) is then applied to each flower’s point cloud, extracting the three principal vectors that describe its orientation. The whole workflow is summarized in Figure 7.

For pose estimation, the flowers in the input image are segmented by the SAM deep learning method. For each flower of interest, the SAM binary segmentation mask is used to extract a point cloud. Once the point cloud is extracted, the PCA algorithm is applied to identify the vector components describing the point cloud distribution. The pose of the flower is determined from the PCA axes (Figure 7). Notably, while the latter part of the workflow is similar to the work of Li et al. [9], our method introduces a zero-shot approach, eliminating the need for any model retraining or transfer.

#### 2.2.3. Plucking Point Estimation

**Figure 8:**
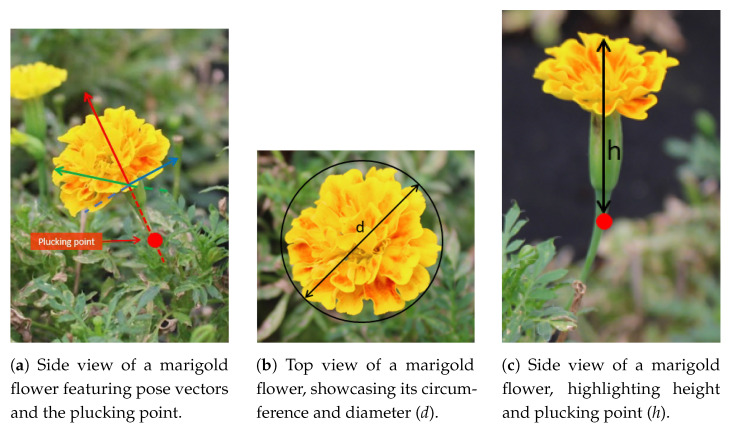
Marigold flowers: side view with pose vectors, top view with circumference and diameter, and height perspective.

Following pose estimation, the vision framework includes a module designed to estimate the optimal plucking point of the flower. This point is the location along the flower stem where the robotic arm’s end-effector should cut to detach the flower from the plant. In manual harvesting, flowers are typically plucked just below the calyx. However, identifying this point through pattern recognition is not feasible due to occlusion by the flower or surrounding leaves. Additionally, using 3D matching is impractical because of the complex and varied morphology of flowers; this method is typically applied to objects with more consistent shapes. The procedure is exemplified in Figure 8a.

The optimal plucking point aligns with the extension of the flower’s vertical component (Figure 8a). Hence, given the pose of the flowers defined by the three position vectors previously calculated, the main challenge is to determine the appropriate distance from the top of the flower (measured by Zed RGB-D cameras) down to the plucking point. This distance is denoted as *h* (Figure 8c). Apparently, the problem has not been directly addressed in the literature. Here, we propose to estimate the optimal *h* by defining a numerical relationship between the diameter of the flower (Figure 8b) and the distance from the top of the flower down to the plucking point (Figure 8c).

To address this relationship, we collected a set of 300 flowers, evenly distributed among the three types of flowers of interest. These flowers were collected and meticulously measured using a caliper. This measurement dataset was developed during the second data acquisition campaign, considering the collection of flowers from the rows where images were captured.

## 3. Results and Discussion

### 3.1. 2D Detection

Notably, the detection component is the only aspect that underwent actual training and evaluation. Indeed, the resulting model, FLOLO, is derived from YOLOv5 large after two fine-tuning steps. Initially, we applied preliminary fine-tuning on the D0 dataset, starting with the weights of YOLOv5l pre-trained on the COCO dataset (D0-FLOLO). Subsequently, final fine-tuning was conducted using the custom FloraDet dataset. Both training phases followed the standard parameters provided by YOLO. Both fine-tuning processes were carried out for 300 epochs, utilizing a learning rate of 0.01, a momentum of 0.937, and a weight decay of 0.005 applied to the SGD optimizer. The standard YOLO training loss function, covering locality loss, confidence loss, and classification loss, was employed. Regarding the data, in both fine-tuning processes, the respective datasets were split into 75% for training, 15% for testing, and the remaining 10% for validation. The optimal models were chosen based on the epoch where the model achieved the highest performance on the validation set. A summary of the trained models, training sets, starting weights, and performance metrics on the test set are schematized in Table 3.

**Figure 9:**
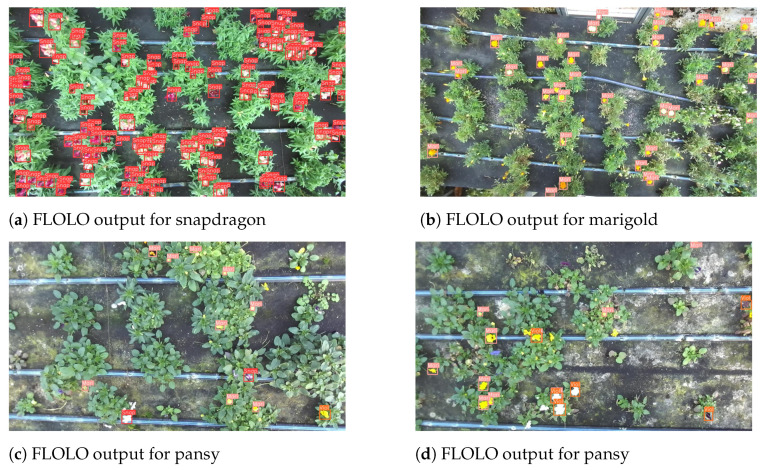
FLOLO outputs for different flowers: (**a**) snapdragon, (**b**) marigold, (**c**) viola (1st view), (**d**) viola (2nd view).

**Figure 10:**
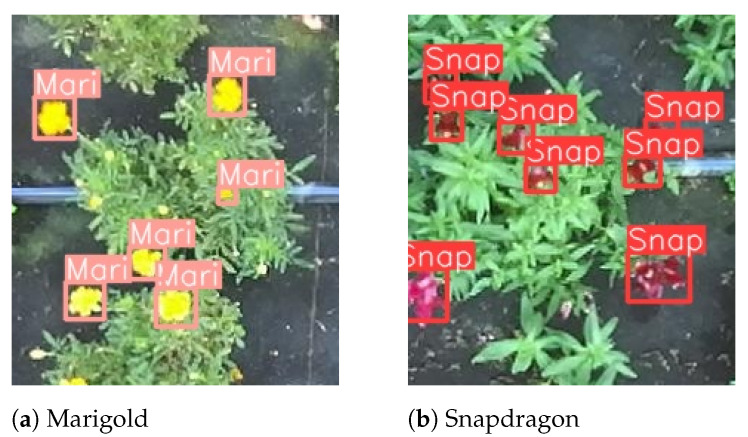
Cropped close-up output from the preceding images (Mari = marigold, Snap = snapdragon). Not-ready-to-be-picked flowers are correctly not detected.

Overall, the three metrics (mAP, precision, and recall, Table 3), demonstrate that FLOLO performs well in detecting flowers, achieving a strong balance between precision and recall. Specifically, the model’s output on test images highlights its effectiveness in detection but reveals occasional challenges in classifying pansies (Figure 9d). This difficulty may be attributed to the underrepresentation of pansies in the dataset, as well as the natural variability within the species, which includes two distinct types and varieties. It is also important to note that the key performance metric, the object detection error on the validation set, scored a low value of 0.045, indicating the model’s success in accurately identifying and localizing ripe flowers. A closer look at the detection performance is provided in Figure 10.

### 3.2. 3D Localization and Pose Estimation

**Figure 11:**
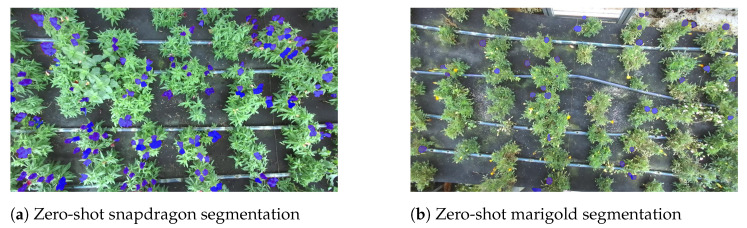

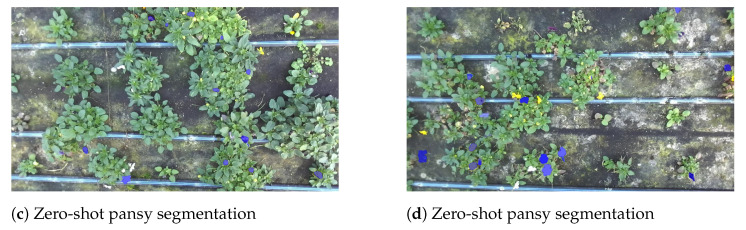
SAM zero-shot segmentation for different flowers: (**a**) snapdragon, (**b**) marigold, (**c**) pansy (1st view), (**d**) pansy (2nd view).

As mentioned in Section 2.2.2 and Section 2.2.3, the 3D localization module was executed without any training, capitalizing on the remarkable zero-shot capabilities of SAM. In the case of segmentation masks comprising separated polygons for the same prompt bounding box, since each bounding box contains only one flower, some of these polygons need to be discarded. To this end, the center of each polygon is extracted, and only the mask with the center point closest to the center of the bounding box is selected. This refined mask is then employed to filter out pixels from the 3D point cloud that have not been identified as detected flowers. An example of flower segmentation is illustrated in Figure 11.

**Figure 12:**
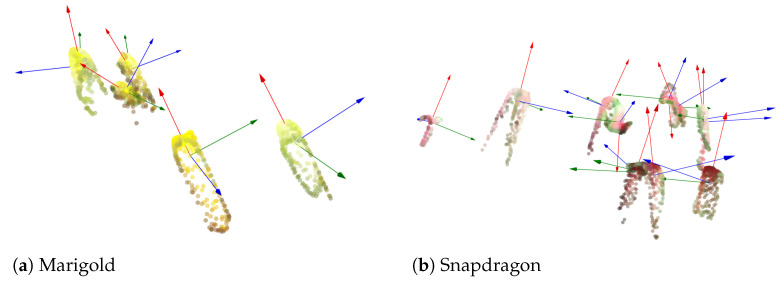
Isolated point clouds of (**a**) marigold and (**b**) snapdragon flowers and the perspectives vector component. For a closer view, see Figure 15, which also shows the estimated plucking points for each flower.

Subsequently, using the segmentation binary mask, only the coordinates of points belonging exclusively to the flowers in the point cloud are isolated, and the vector component of each flower’s point cloud is computed by PCA. An example of the process applied to estimating the pose for each flower to be subsequently utilized to determine the optimal plucking point is shown in Figure 12. Pose estimation examples are presented for smaller frames of marigold and snapdragon flowers, previously displayed in Figure 9.

The high density of flowers makes it possible that some flowers may overlap. In such cases, the pose is estimated only for the flower with a complete point cloud. As mentioned in the introduction, since flowers are harvested daily, or even multiple times a day, and the picking order is random: once the top flower is picked, the flower beneath it becomes fully exposed and ready to be harvested next time.

### 3.3. Plucking Point Estimation

**Figure 13:**
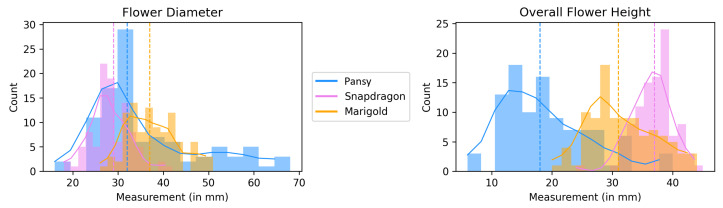
Distribution plots for total flower diameter (**left**) and height (**right**). Dotted vertical lines indicate medians, horizontal solid line represents a smooth approximation of the distribution.

In order to define a plucking point estimation procedure, we developed an automatic analysis of the flower structures. The distribution of flower measurements is presented in Figure 13 for flower diameter *d* and *h* overall flower height, as shown in Figure 8b,c, respectively.

The distribution of measured diameters for pansy flowers exhibits a prolonged right tail. This phenomenon is attributed to the significant size difference between the two pansy species under consideration (Carneval and Sorbet), with one being substantially larger than the other. The dataset appears to under-represent the larger pansy due to recent harvesting, contributing to the presence of a right tail.

By analyzing the plot of overall flower height, the right tail is less pronounced for pansy flowers, suggesting reduced variability in flower height compared to flower diameter. For the purpose of plucking point estimation, relying on a predefined fixed value based on median values is unsuitable, as too many flowers will fall to the right of this value. Consequently, the estimated plucking point would be situated within the calyx, compromising the flower’s integrity during picking. Therefore, it is preferable to cautiously overestimate the optimal plucking point within a certain margin.

**Figure 14:**
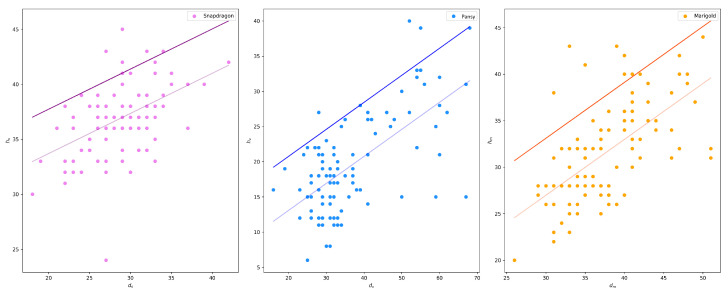
Scatter plot of flower diameter against total flower height. Light solid lines depict the linear regression line, while darker lines represent the 85% upper boundaries.

The potential relationship between *h* and *d* was assessed for each flower variety through linear regression (Figure 14) using the scikit-learn 1.3.2 [31] package in a python environment. Moreover, 80% of the measurements were used to investigate this relation and the remaining 20% to test it. While the regression line generally fits the distribution well, many points lie on top of the line, indicating potential damage to the flower if this inference will be relied upon for cutting. To mitigate this drawback, the plucking point estimation module does not use the regression line for estimating h given d. Instead, a translated line corresponding to the upper boundary of the 85% confidence interval is selected. Table 4 lists both the regression line and the exploited upper boundary equations for each flower type. The 85% threshold was chosen to balance the trade-off between overestimating the cutting point and minimizing damage to the flowers. Indeed, in the test set, only the *h* of one flower for snapdragon and pansy respectively and two for marigold were underestimated. Further field testing is needed for fine-tuning this value.

**Figure 15:**
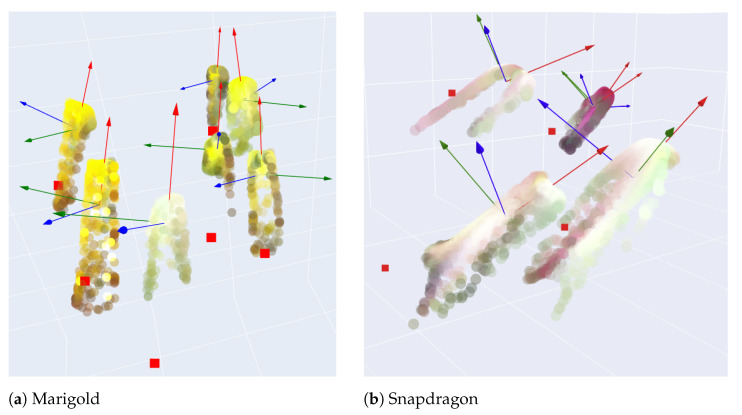
Isolated point clouds of (**a**) marigold and (**b**) snapdragon flowers and the respective vector components (red, green and blue vectors) and estimated plucking point (red squares).

In the inference phase, the framework inputs the binary mask, flower point cloud, pose estimation, and the equations, as estimated in Table 4. In the case of different flower species, an additional regression model can be estimated. The center point of the flower is found among the top points of the 3D cloud. Given the binary mask, the flower diameter is calculated by estimating the smallest convex polygon enclosing the points. The center point is defined as the point within the polygon that has the maximum distance to the boundary of the polygon. This process calculates *d*. The next step is estimating *h* through the previously calculated functions. Finally, with a flower pose, the center point is translated down along the main vertical vector by the calculated *h*. This point represents the estimated optimal plucking point and is denoted by the red square in Figure 15a. Additionally, the figure shows the final result of the entire framework for a sample of marigold and snapdragon flowers. The pose vectors, red, green, and blue, are displayed, with the red vector representing the main axis that describes the flower’s pose. Comparing the two figures demonstrates that the pose is successfully estimated for both upward-facing flowers (marigold) and tilted flowers (snapdragon).

### 3.4. Speed Performance Evaluation

While this work is primarily a proof of principle, it is interesting to examine the speed performance of the overall process for its prospective use as an AI tool for on-field application. We report in Table 5 the average time, in seconds, required to process the validation images with a varying number of flowers for two varieties of interest, for a total of more than 1400 flowers.

The most computationally intensive step pertains to the isolation of 3D points. This is primarily due to the substantial search space, comprising about 921,000 points in this configuration. However, this process can be optimized in two ways: first, by undersampling the search space, and second, by implementing an initial filtering step, such as considering only points near the bounding boxes. Further speed improvements can be achieved by optimizing the code through vectorizing certain operations, potentially reducing the computational complexity to sublinear levels.

The key metric from the perspective of real-field implementation is the total time required to process a single flower. In this study, the processing time was 1.064 s for marigold flowers and 0.98 s for snapdragons, which are comparable to times reported in the literature, such as those found in the work of Li et al. [9].

In conclusion, it can be asserted that, with adjustments to the pipeline and addressing the highlighted critical points, the overall framework could also be utilized for in-field live inference validation steps.

### 3.5. Advantages, Limitations, and Future Perspectives

#### 3.5.1. Advantages

The framework demonstrates robustness and adaptability across various species and varieties of edible flowers. Through the integration of AI tools utilizing few-shot, zero-shot, and standard machine learning techniques, we have minimized the need for extensive data collection and annotation, streamlining the development process. The successful detection, pose estimation, and plucking point estimation capabilities of our framework lay the foundation for automated robotic harvesting, offering significant efficiency gains for growers.

#### 3.5.2. Limitations

One limitation of this work is the assessment of the pose estimation module. Indeed, to fully evaluate pose estimation accuracy, a functional robotic picking system is required, as it provides a reliable ground truth for performance evaluation. This aspect will be addressed in future work when a robotic arm is integrated into the picking tests. Additionally, factors such as flower occlusion, environmental variability, and seasonal fluctuations in flowering may influence detection accuracy and system performance under dynamic conditions.

#### 3.5.3. Future Perspectives

Moving forward, our focus will be on refining the framework to address its computational demands, with the aim of making it more suitable for practical deployment in real-world scenarios. Additionally, we plan to integrate the framework into a robotic arm for automated flower harvesting, facilitating in-field validation of its performance under varying environmental conditions. This iterative process will involve rigorous testing and validation to ensure the framework’s effectiveness and reliability in agricultural settings.

## 4. Conclusions

The primary objective of this study was to assess the viability of an AI-based vision framework for the robotic harvesting of edible flowers, specifically aiming to evaluate its performance on different species and varieties of flowers. Additionally, this work examined the capabilities of foundational models and state-of-the-art architectures to minimize computational resource requirements and overcome data availability constraints. The (FloralAI) framework can be applied across different species and varieties of flowers, indicating the robustness and adaptability of this approach to various crops. The application of general-purpose and foundation models such as YOLOv5 and SAM enabled the use of a robust approach based on tuning on public data and zero—shot application of data from the field. The result suggests that with minor fine-tuning, it is possible to extend the same approach to various scenarios.

Next steps could also see the application of novel architectures, such as Fast Segment Anything [32], especially to directly segment only ripe flowers. Additionally, refining the framework through code optimization to reduce complexity and speed up computationally demanding processes will be essential. This approach could lead to more reliable and computationally efficient results, thereby supporting practical edge computing in real-world scenarios. Moreover, future data campaigns will be conducted to enrich the dataset and address the under-representation of pansies.

Furthermore, the proposed framework also holds potential for future annotation processes. Our method can serve as a ground truth annotation engine supporting the development of upcoming architectures. The availability of high-quality annotations is a significant barrier to training efficient neural networks, and the methods described here can provide reliable ground truth for training more complex architectures.

To extend this approach to other flowers and similar crops, a deeper understanding of the crop canopy is essential for developing a generalized method for pose and plucking point estimation. Different flowers do not always exhibit the same manner of attachment to the stem, so it would be beneficial to explore whether a framework can be structured to accommodate this variability.

Current efforts are focused on implementing a path planning algorithm specifically designed for the robotic harvesting of flowers identified by the FloralAI framework. The next phase will facilitate in-field empirical validation tests of the framework and its application in managing robotic harvesting based on our pose and plucking point estimation modules. 

## Figures and Tables

**Table 1:** Structure of the FloraDet dataset. The number of collected images and number of flowers from those images in the two data campaigns are listed in the three varieties, subdivided on Month sampling.

	Items	Pansy	Marigold	Snapdragon
July	Images	-	25	13
	Flowers	-	854	762
November	Images	64	21	11
	Flowers	375	401	51

**Table 2:** Common name, ImageNet synset code for ImageNet, number of images, and flowers constituting the support D0 dataset from a public data source. Pansy data were collected from Kaggle without a specific synset reference.

Common Name	ImageNet Synset	Number of Images	Number of Flowers
Sunflower	n11978233	245	294
Calla lily	n11793779	179	233
Cornflower	n11947802	126	146
Dahlia	n11960245	172	191
Strawflower	n11980318	172	242
Coneflower	n11962272	139	159
Pansy	-	232	905

**Table 3:** Details on D0-FLOLO and FLOLO architectures. Main performance metrics, starting weights, and training sets for the two fine-tuned architectures.

Model Name	Training Set	Starting Weights	Best Epochs	mAP 0.5	Precision	Recall	Det. Val. Error
D0-FLOLO	D0	YOLOv5-large	284	0.97	0.96	0.93	0.0039
FLOLO	FloraDet	D0-FLOLO	229	0.68	0.67	0.68	0.045

**Table 4:** Equations for the linear regression and the respective upper boundary for each of the different flower types.

	Pansy	Snapdragon	Marigold
Linear Regression	$h_{v}=0.36d_{v}+26.33$	$h_{s}=0.38d_{s}+5.33$	$h_{m}=0.66d_{m}+7.10$
Upper boundary	$h_{v}=0.36d_{v}+43.20$	$h_{s}=0.38d_{s}+34.57$	$h_{m}=0.66d_{m}+37.91$

**Table 5:** Average processing inference time (seconds) for each step of the AI framework on an NVIDIA GeForce RTX 3090. The presented averages are computed based on the analysis of 10 images for each of the two flower types and including a total of 457 marigold and 975 snapdragons flowers.

	Detection	Localization			Estimation		Total	Time
	YOLO	SAM	Clean Masks	Isolate 3D Points	Pose (PCA)	Plucking Points	Time	per Flower
Marigold	0.285	4.37	0.08	42.36	1.44	1.48	48.35	1.064
Snapdragon	0.293	4.36	0.165	89.90	5.76	5.87	95.99	0.98

## Data Availability

The raw data supporting the conclusions of this article will be made available by the authors on request.
